# Identifying Biomarkers for Biological Age: Geroscience and the ICFSR Task Force

**DOI:** 10.14283/jfa.2021.5

**Published:** 2021-03-08

**Authors:** Nathan K. Lebrasseur, R. de Cabo, R. Fielding, L. Ferrucci, L. Rodriguez-Manas, J. Viña, B. Vellas

**Affiliations:** 1 Robert and Arlene Kogod Center on Aging and Department of Physical Medicine and Rehabilitation, Mayo Clinic, Rochester, MN USA; 2 Translational Gerontology Branch, National Institute on Aging, Baltimore, MD USA; 3 Tufts University, Boston, MA USA; 4 Intramural Research Program, National Institute on Aging, Baltimore, MD USA; 5 Servicio de Geriatría, Hospital Universitario de Getafe, Toledo, Spain; 6 Freshage Research Group, Department of Physiology, Faculty of Medicine, University of Valencia, CIBERFES-ISCIII, INCLIVA, 46010 Valencia, Spain; 7 Gerontopole, INSERM U1027, Alzheimer’s Disease Research and Clinical Center, Toulouse University Hospital, Toulouse, France

**Keywords:** Aging, frailty, resilience, hallmarks of aging, translational research

## Abstract

The International Conference on Frailty and Sarcopenia Research Task Force met in March 2020, in the shadow of the COVID-19 pandemic, to discuss strategies for advancing the interdisciplinary field of geroscience. Geroscience explores biological mechanisms of aging as targets for intervention that may delay the physiological consequences of aging, maintain function, and prevent frailty and disability. Priorities for clinical practice and research include identifying and validating a range of biomarkers of the hallmarks of aging. Potential biomarkers discussed included markers of mitochondrial dysfunction, proteostasis, stem cell dysfunction, nutrient sensing, genomic instability, telomere dysfunction, cellular senescence, and epigenetic changes. The FRAILOMICS initiative is exploring many of these through various omics studies. Translating this knowledge into new therapies is being addressed by the U.S. National Institute on Aging Translational Gerontology Branch. Research gaps identified by the Task Force include the need for improved cellular and animal models as well as more reliable and sensitive measures.

## Introduction

The International Conference on Frailty and Sarcopenia Research (ICFSR) Task Force met in Toulouse, France on March 10, 2020 to discuss geroscience. The timing could not have been more prescient: On the following day, March 11, the World Health Organization (WHO) declared the COVID-19 outbreak a pandemic (1). The disease, caused by a virus known as SARS-CoV2, had at that point already claimed the lives of more than 4,000 people in 114 countries, with the risk of morbidity and mortality especially elevated in older people and those with underlying medical conditions (2).

Why older adults are particularly vulnerable to this novel virus remains poorly understood. Age-related physiological changes including immune senescence, the high incidence of chronic illnesses, and frailty may decrease resilience and increase susceptibility to cardiovascular, pulmonary, and infectious diseases (3). Older adults may also present atypically, further complicating diagnosis and treatment (4).

Targeting the biology of aging to prevent and treat aging-associated diseases and geriatric syndromes as a group represents a fundamentally different approach to extending human health. Historically, drug development efforts have centered on evidence-based risk factors identified through epidemiological studies and specific molecular alterations that contribute to singular diseases. This approach has had marked success but has also revealed that interventions for a specific disease, whether it be heart disease, cancer, Alzheimer’s Disease, pneumonia, or COVID-19, have a limited impact on the emergence of the multitude of other age-related conditions. The promise and potential payoff of interventions for aging and compressing morbidity is high; however, a more in-depth understanding of the underlying biology is needed. In response, the interdisciplinary field of geroscience has emerged to explore biological mechanisms of aging and determine how these mechanisms lead to the vast collection of age-related chronic diseases and geriatric syndromes, including sarcopenia and frailty (5–8). Geroscience approaches are clearly and urgently needed as well to better understand the susceptibility of older adults to acute challenges, such as COVID-19, and develop novel treatments for the most vulnerable individuals, including frail older adults (3).

Specialties represented in the ICFSR Task Force play a critical role in advancing geroscience because they are expert in 1) the discovery and quantification of the biological mechanisms of aging; 2) the study of aging and aging-related diseases in preclinical models, and; 3) the design and execution of clinical trials of testing interventions (exercise, dietary modifications, drugs, and combinations thereof) to optimize the health and function or resilience of multiple physiological systems. Indeed, a multidisciplinary approach is necessary to expedite the translation of newly discovered therapies to clinical application.

## What is healthy aging?

The WHO introduced the concept that healthy aging and disease prevention hinges on preventing declines in intrinsic capacity — a composite of physical and mental capacities that peak in early adulthood and tend to decline in later years. WHO developed a model for integrated care of older people (ICOPE) that focuses on maintaining intrinsic capacity through the adoption of a healthy lifestyle (9).

As individuals age, they transition between robustness to frailty, defined as increased vulnerability to endogenous and exogenous stressors and a decline in physiological reserve and function across multiple organ systems. Thus, frailty and intrinsic capacity represent distinct but related concepts, both with similar physiological underpinnings (10). Physiological mechanisms of resilience and reserve further impact the capacity of an individual to overcome adverse events (11). Geroscience resides at the intersection of these concepts (12).

## Biological hallmarks of aging

Geroscience assumes that aging itself, defined as the accumulation of diverse forms of molecular and cellular damage and repair, ultimately drives the increased risk of chronic diseases among older people (13). López-Otin and colleagues proposed nine distinct yet interrelated forms, or “hallmarks”, of aging: genomic instability, telomere attrition, epigenetic alterations, loss of proteostasis, deregulated nutrient sensing, mitochondrial dysfunction, cellular senescence, stem cell exhaustion, and altered intercellular communication (14). These hallmarks have been incorporated into an emerging view of geroscience that resulted in the creation of a trans-National Institutes of Health (NIH) Geroscience Interest Group, GSIG (15).

Together, the biological mechanisms progressively result in loss of cellular homeostasis, dysregulation across multiple physiological systems and, ultimately, disease, disability, and death. Both the accumulation and the repair of aging hallmarks are strongly influenced by behavior, the environment, and genetics, resulting in substantial variation among individuals. This has led to the important concept that biological age differs from chronological age. Biological age, in contrast to chronological age, is difficult to quantify, which has led to inconsistent definitions in the literature. Herein, approaches to measure aging hallmarks are presented as a means to better define biological age of cells, tissues, and, ultimately, organisms.

One of the longest longitudinal studies of normative human aging, the Baltimore Longitudinal Study of Aging (BLSA), has been running for more than 60 years. The BLSA collected multidomain clinical and functional data from participants with increasing frequency as they aged: every 4 years for those under age 60 increasing to every year for those over age 80. Using these data, the investigative team recently proposed a roadmap to build a phenotypic metric of aging, which by systematically characterizing the continuum of aging in an individual, could advance understanding of the kinetics of aging, as well as discovery and development of effective interventions. The framework encompasses four domains: body composition, energetics, homeostatic mechanisms, and neurodegeneration/neuroplasticity (16).

Scientists in the NIA Intramural Research Program are also conducting a Study of Longitudinal Aging in Mice (SLAM) to better understand whether discoveries in mice can be translated to humans and which aging phenotypes share or do not share common traits in order to fine tune interventions to translatable outcomes. They are conducting the study in two strains of mice of both sexes, selecting the C57BL6/J and the UM-HET3 mice to better recapitulate both the genetic homogeneity of most aging studies and the heterogeneity found in humans respectively. SLAM investigators are conducting a broad range of clinically relevant assessments over time and across multiple domains. To assess frailty, they will apply two newly developed tools: the mouse clinical frailty index and mouse frailty phenotype assessment (17). For example, at 3-month intervals, they assess gait speed, fasting blood glucose, energetic cost on a metabolic treadmill, and frailty. They also perform imaging tests such as magnetic resonance imaging (MRI), nuclear magnetic resonance (NMR), and micro-computed tomography (micro-CT) to obtain organ images and spectra, body composition, and bone mass changes with age.

## Implementing biomarkers of aging in clinical research

Metrics of aging span multiple domains and include biological hallmarks, organ impairments (e.g., muscle weakness), functional limitations (e.g., slow gait speed), and disease and deficit accumulation (e.g., frailty). Undoubtedly, as people age, the onset and progression of decline within each domain differs; and understanding whether biomarkers of these different domain are mechanistically connected and exploring temporal relationship between domains is critical to translate this science into effective interventions. Based on the geroscience premise that aging itself is the driver of the majority of chronic diseases and geriatric syndromes, quantifiable indicators or “biomarkers” of biological age would be of significant utility to target individuals who are experiencing accelerated aging as well tracking the effectiveness of interventions aimed at slowing down the aging process.

For clinical research, biomarkers of aging could enable identification of persons appropriate for and, potentially, most responsive to interventions targeting the biology of aging. In such trials, biomarkers may be used to verify target engagement and also serve as informative surrogate endpoints that may change well before clinical outcomes (e.g., frailty measures (18)). In clinical practice, biomarkers may help providers discern between chronological and biological age and, in turn, serve as determinants of risk and guide clinical decision making (e.g., medical versus surgical management of a condition). Implementing laboratory biomarkers of aging in clinical research and practice, however, will first require demonstrating that they can be reliably measured in blood or other accessible tissues and reflect clinical manifestations of aging. Recently, a candidate panel of senescence biomarkers was developed based on the secretome of senescent human endothelial cells, fibroblasts, preadipocytes, epithelial cells, and myoblasts in vitro. In older adults undergoing surgery, the senescence biomarkers were shown to correlate with chronological age and biological age, as defined by the frailty index, and to predict adverse events such as surgical complications and rehospitalizations (19).

Table 1 lists some potential biomarkers of the hallmarks of aging as well as the biological matrix in which they could be assessed.

**Table 1:** Potential Biomarkers of the Hallmarks of Aging (courtesy of Tamara Tchkonia and Nathan LeBrasseur)

**Hallmark**	**Biomarker Measures**	**Biological Matrix**
Mitochondrial dysfunction	Mitochondrial function/respiration	PBMCs
	Mitochondrial volume, number, shape	Tissue biopsy
	Markers of biogenesis	mtDNA
	mtDNA copy number, mutations, haplotypes	
	NAD+ metabolites	
	Sirtuins	
Loss of proteostasis	Autophagy markers and flux	Blood
	Chaperone proteins	Tissue biopsy
	Advanced glycation end products (AGEs)	Live cells
	Protein aggregates	CSF
Stem cell dysfunction	Replication/differentiation potential	Blood
	Tissue regeneration	Tissue biopsy Live cells
Nutrient sensing	IGF-1 pathway	Blood
	mTOR signaling	Tissue biopsy
Genomic Instability	Single-cell/NGS, SNP analyses	Blood
	DNA repair	PBMCs
	Measures of DNA modifications	Tissue biopsy
	Long interspersed nuclear elements (LINEs)	Live cells
	Reverse transcriptase	
Telomere dysfunction	Telomere length	Blood
	Markers of DNA damage response	PBMCs
	Telomerase activity	Tissue biopsy
	Telomere-associated foci	
Cellular senescence	p16, p21, p53	Blood
	Histological marks (SABG, TAFs, SADs)	PBMCs
	SASP products, miRNA, circulating mtDNA	Tissue biopsy
	Extracellular vesicles and microvesicles	CSF
	Circulating p16Ink4A + CD3+ T cells	Urine
Epigenetic changes	Methylation	Blood
	Histone acetylation	PBMCs
	Non-coding RNAs	Tissue biopsy Swab

Omics-based laboratory biomarkers have also been investigated in the FRAILOMIC initiative (20,21), an international consortium funded by the European Commission that aims to develop omics-based clinical instruments to assess frailty and predict the risk of frailty and subsequent disability. The consortium is analyzing data from four European cohorts: InCHIATIi (Tuscany, Italy), AMI (Gironde, France), the Three-City (3-C)Study (Bordeaux, France) (3C), and Toledo Study for Healthy Aging (TSHA, Toledo, Spain). The wide range of potential biomarkers investigated in the exploratory phase of this initiative is shown in Table 2.

**Table 2:** Exploratory phase biomarkers (courtesy of Prof. L. Rodriguez Mañas)

Genomics and transcriptomics	Sistemas Genómicos	Genotyping of 256 polymorphism in candidate genes SNPs Expression study of candidate genes: IL4, IL7, IL17A, FASLG, MTOR, BCL2L1, FAS, OAZ1_HK, PMAIP1, IL10, NFE2L2, IL6, IFNG, TGFB1, PPARGC1A, TP53, PPARD, B2M_HK
	LifeLength	Telomere length
	Evercyte	96 Circulating miRNA (Aging/Senescence/Inflammaging/Longevity, Bone metabolism, musculoskeletal function, and fracture risk, Cardiovascular Disease)
	Sermas	Expression studies target genes for hypoxia inducible factors-HIF and ACE2, ARG2, CXCL10, EGLN3, EPAS1, MAS1, HMOX2, HIF1A, HIF3A, IL10, IL6, KDR, NOS2, NFE2L2, PTGS2, SOD2, SIRT1, CXCL12, TXNRD1, CYP27B1, VDR
Proteomics	Innsbruck	Serum concentration of secreted proteins from senescent endothelial cells. JAG1, IGFBP6, VERSICAN
	Cardiff	Plasma levels of glycated proteins, its soluble receptor and cognitive performance BM. CCL11, RAGE
	Sermas	Serum levels of HIF
	DIFE	Oxidized proteins: Protein.carbonyls, 3-Nitrotyrosine
	Mosaiques	Urinary proteome analysis
Metabolomics	U. Valencia	Metabolites: FFAA-CH2 & n.alloisoleucine.2, hydroxyvalerate & threonine & lactate, lysine/valine, CH2-isoleucine and CH-CH2-CH2
Classical non-omics biomarkers	Parma	Determination of traditional BM related to frailty: SuPAR, Pro.BNP, Troponin T, VCAM.1, ICAM-1, MMP-9, MMP-11, ACTIVIN-A, ADIPONECTIN, MYOSTATIN, GALECTIN-3, PCT, ESTRADIOL, A.N.A
	U. Valencia	BM of lypoperoxidation MDA, Polypeptides in urine
	DIFE	Fat-soluble micronutrients, 25 Hydroxyvitamin D D3 and D2 separately), Sum_lutein_zeaxanthin Beta, alpha_carotene, Retinol, protein_carbonyls, 3-Nitrotyrosine, Tocopherol, -Tocopherol, -Cryptoxanthin, Lycopene, Lutein/Zeaxanthin

FRAILomic studies thus far have shown that biomarkers of frailty change according to clinical characteristics of participants, suggesting the existence of different clinical phenotypes of frailty. For example, omics biomarkers may be associated with disability, sarcopenia or other organ-specific diseases, and vary by sex, ethnicity, and race. Lab biomarkers appear to be modestly associated with classical biomarkers such as biomarkers of inflammation, hormonal changes, and glucose dysregulation (23), particularly among individuals with disability.

For example, in one study of FRAILomic participants, serum levels of the soluble receptor for advanced glycation end-products (sRAGE) was shown to be an independent predictor of mortality in frail individuals, suggesting that sRAGE levels may be useful for prognostic assessment and treatment stratification (24). Another study demonstrated that frail participants had higher plasma levels of 3-methylhistidine (3-MH) and higher ratios of 3-MH to creatine and 3-MH to estimated glomerular filtration rate, suggesting that these markers may be useful in identifying individuals at risk of frailty Finally, in this same regard, fat-soluble vitamins and carotenoids are biomarkers of frailty status (robust, pre-frail, frail) (26) but do not predict the risk of becoming frail (27)

Future studies of biomarkers of aging and their cross-sectional and longitudinal relationships with parameters of function (e.g., physical, cognitive, cardiovascular, pulmonary, renal metabolic, immune, and sensory) and resilience (e.g., to infection and consequences of SARS-CoV2) affected by advancing age are warranted. Longitudinal studies promise to provide greater insights into rates of biological aging and, potentially, in the context of clinical trials, the extent to which the molecular and cellular effects of aging can be attenuated and/or reversed. As novel interventions targeting the biology of aging emerge, biomarkers of aging will facilitate their testing and development.

## Translation — developing agents that target fundamental aging processes

The Translational Gerontology Branch (TGB) at the National Institute on Aging (NIA) is part of the NIA’s intramural research program. Research conducted in TGB labs ranges from drug discovery using a variety of in vivo and in vitro models to clinical and longitudinal studies. For example, TGB researchers are studying the cellular and molecular mechanisms underlying aging, diseases of aging, and longevity, including the hallmarks mentioned earlier. Clinical studies have explored domains of the aging phenotype such as changes in body composition, energy imbalance, homeostatic dysregulation, and neurodegeneration and the impact of those changes on disease susceptibility, reduced functional reserve, impaired stress response and healing capacity, impaired physical function, disability, and dementia. The NIA also supported establishment of the Translational Geroscience Network (TGN) to develop, implement, and test standard operating procedures for translational early phase trials of agents that target fundamental aging processes and to select, optimize, and validate ancillary measures of fundamental aging processes for use across all trials (R33 AG061456). TGN provides statistical and data management support and has established a biobanking and repository network.

## Conclusions

Healthy aging involves both delaying the physiological consequences of aging and maintaining functioning as aging progresses. Interventions thus need to focus on preventing frailty and disability. To develop effective and feasible interventions for healthy aging, whether drugs, exercise, diet, or combinations thereof, will require a deeper understanding of the mechanisms of aging as well as identifying biomarkers that track with biological, not simply chronological age, and predict when an individual is approaching a tipping point and nearing a threshold of irreversible decline.

The pathway to these biomarkers is through the interdisciplinary field of geroscience, which seeks to define the biological mechanisms of aging that give rise to age-related diseases and disorders and to identify targets that may be amenable to different kinds of interventions (15). Developing these biomarkers will require improved cellular and animal models and the capacity to translate discoveries from those models into humans. Also required will be reliable and sensitive measures to assess the hallmarks of aging, for example, assessments of mitochondrial dysfunction, and the impact of interventions on these biological mechanisms and, in turn, the health and functioning of older adults.

The interdisciplinarity of geroscience will be essential to define the complex interactions of the multiple biological, physiological, and behavioral pathways that contribute to age-related declines in health. Interdisciplinarity will also ensure that advances in geroscience are applied to other biomedical disciplines such as neuroscience and cardiology.

## Notes

*Ethical Standards:* None.
